# Supplementary material for: Bisphenol A Exposure Changes the Transcriptomic and Proteomic Dynamics of Human Retinoblastoma Y79 Cells
Source: Genes (Basel). 2021 Feb 11;12(2):264. doi: 10.3390/genes12020264 (PMC7918513; doi:10.3390/genes12020264)
Supplement: Supplementary file 1 [file genes-12-00264-s001.zip › genes-1096014 supplementary updated/genes-1096014-supplementary figures.docx]

Article

Bisphenol A exposure changes the transcriptomic and proteomic dynamics of human retinoblastoma Y79 cells

Chul-Hong Kim^1,2^, Mi Jin Kim^1,2^, Jinhong Park^1^, Jinho Kim^1^, Ji-Young Kim^1^, Mi-Jin An^1^, Geun-Seup Shin^1^, Hyun-Min Lee^1^, Jung-Woong Kim^1,^*

^1^ Department of Life Science, Chung-Ang University, Seoul 06974, South Korea; 01031e70067@gmail.com (C.-H.K.); mjkim831025@gmail.com (M.J.K.); shoopjh@cau.ac.kr (J. P.); jinhokim07@cau.ac.kr (J. K.); jykim@cau.ac.kr (J.-Y.K.); dksalwls333@gmail.com (M.-J.A.); rjstjq89@naver.com (G.-S.S.); lhmscb@naver.com (H.-M.L.),

* Correspondence: jungkim@cau.ac.kr; Tel.: +82-2-820-6682, Fax: +82-2-815-6682

|  |
| --- |

**Figure S1.** Cytotoxic effect of BPA on Y79 retinoblastoma cells. Cells were treated with the indicated concentration of BPA for 48 h. **(a)** Cell viability was measured using the MTS assay. Cells were treated with 0-1000 μM for 24 h or 48 h. Data are represented as the percentage of the values obtained for the DMSO-treated (0.1% v/v) cells used as the treatment control. ‘control’ means untreated naïve cells in the culture media. **(b)** The BPA-treated Y79 cells were immunostained with anti-Ki-67 antibodies. The cells were counted by FACS analysis. **(c)** The BPA-treated Y79 cells were stained with propidium iodide (PI), and the cell cycle was measured using FACS analysis. The percentages of population in the sub-G1, G0/G1, S, and G2/M-phases are represented as the mean ± S.E.M. of three independent experiments (n = 6), each performed in triplicate.

**Figure S2.** The effect of BPA on cell cycle-related protein expression in Y79 retinoblastoma cells. Cells were treated with indicated concentration of heavy metals for 24 or 48 h. Cells were fixed with 1 % PFA and stained with anti-cyclin B1 **(a)** or anti-cyclin D1 **(b)** antibody. The protein expression was analyzed by FACS analysis. The percentages of cyclin B1- or cyclin D1 positive cells are represented as the mean ± S.E.M. of three independent experiments (n = 6), each performed in triplicate. 0.1% DMSO was used as the negative control.

**Figure S3.** Effect of apoptotic cell death upon BPA treatment on Y79 retinoblastoma cells. **(a)** Cells were treated with indicated concentration of BPA for 24 or 48 h, and were double-stained with annexin V-FITC and PI. The proportion of apoptotic cells was assessed by FACS analysis. The scatter plots represent PI (Y-axis) and annexin V-FITC (X-axis). **(b)** After treatment of BPA for 24 or 48 h. Cells were fixed with 1 % PFA for 6 hrs and stained with anti-cleaved caspase-3 antibody. Expression levels of cleaved caspase-3 were analyzed by FACS analysis. Data are represented as the mean ± S.E.M. of three independent experiments (n = 6), each performed in triplicate. 0.1% DMSO was used as the negative control.

**Figure S4.** A visualization of Gene Ontology (GO) annotations using Gorilla tool. Cells were treated 40 μM BPA for 48 h, and then the construction of RNA-sequencing library was performed using 1 μg of isolated total RNA. The dataset for upregulated genes of transcriptome profiling show enriched GO terms of biological processes (a) and molecular function (b). The color indicates the degree of enrichment, from dark orange which means significantly enriched, to white which means not enriched. All was selected for ontogeny choices and the *P*-value < 0.01.

**Figure S5.** The dataset for downregulated genes of transcriptome profiling show enriched GO terms of biological processes **(a)** and molecular function **(b)**. The color indicates the degree of enrichment, from dark orange which means significantly enriched, to white which means not enriched. All was selected for ontogeny choices and the *P*-value < 0.01.

**Figure S6.** Transcriptome dynamics identifies the alternative splicing events in BPA-treated Y79 cells. Differential splicing events in the BPA-treated group shown the clear retained intron (RI) in ABCF3 **(a)**, HNRNPD **(b)** and RELA **(c)** genes. The yellow shadow indicates the retained intron region in BPA-treated Y79 cells.

**Figure S7.** Transcriptome dynamics identifies the alternative splicing events in BPA-treated Y79 cells. Differential splicing events in the BPA-treated group shown the clear retained intron (RI) in HNRNPM **(a)**, FUS **(b)** and RBM3 **(c)** genes. The yellow shadow indicates the retained intron region in BPA-treated Y79 cells.
